# Supplementary material for: Insights into the Structure and Function of TRIP-1, a Newly Identified Member in Calcified Tissues
Source: Biomolecules. 2023 Feb 22;13(3):412. doi: 10.3390/biom13030412 (PMC10046519; doi:10.3390/biom13030412)
Supplement: Supplementary file 1 [file biomolecules-13-00412-s001.zip › Supplementary Materials.pdf]

Supplementary Figures

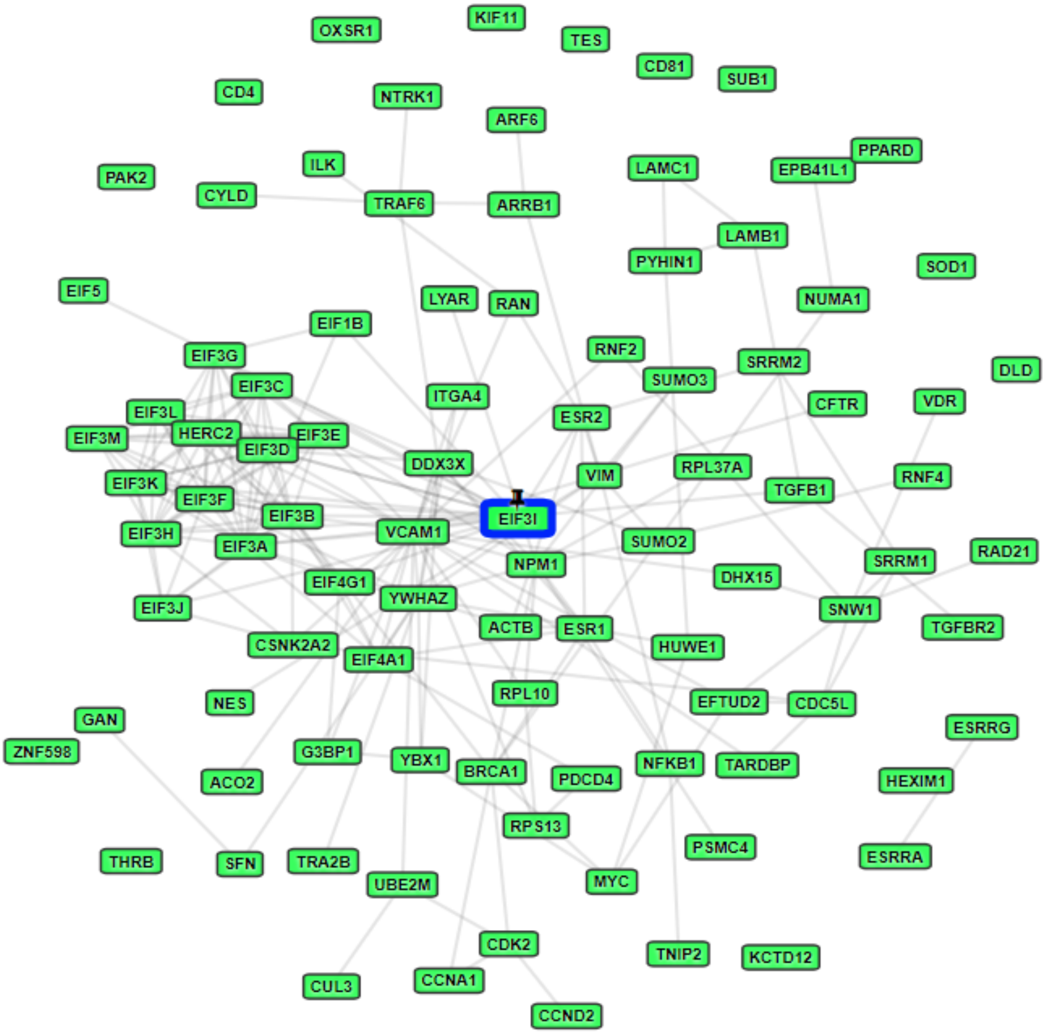

Supplementary Figure S1. Network analysis of the molecular interaction of EIF3i obtained from GPS-Prot

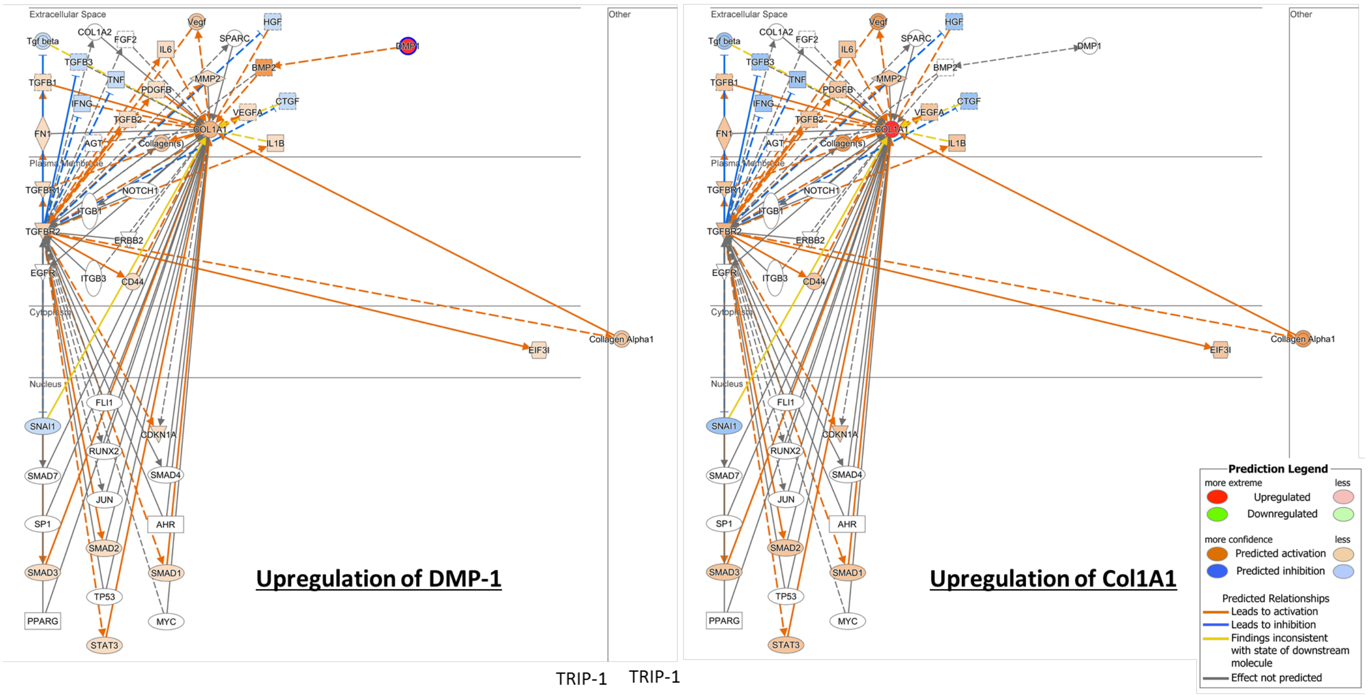

Supplementary Figure S2. Network analysis of IPA generated pathway showing the interaction between DMP-1, Col1A1 and TRIP-1 with TGFBR2 as a connecting node. The interactome on the right and left shows the consequent upstream and downstream molecules when Col1A1 and DMP-1 are enriched *in silico.*

Supplementary Table S1. Table showing the list of proteins identified from MVs of MC3T3 and MC3T3-TRIP-1OE cells. The table is sorted based on protein expression values. (Please find in attached excel sheet).

Supplementary Table S2. List of phosphorylation sites predicted by PhosphoSite Plus®. (Please find in attached excel sheet).

Supplementary Table S3. List of interactors of EIF3i protein obtained from GPS-Prot.

Gene	Forward primer	Reverse primer
**BMP-2**	ACTACCAGAAACGAGTGGGAA	GCATCTGTTCTCGAAAAACCT
**BMP-6**	AGCGACACCACAAAGAGTTCA	GCTGATGCTCCTGTAAGACTTGA
**RUNX-2**	TGGTTACTGTCATGGCGGGTA	TCTCAGATCGTTGAACCTTGCTA
**OCN**	CACTCCTCGCCCTATTGGC	CCCTCCTGCTTGGACACAAAG
**HIF1-A**	CACCACAGGACAGTACAGGAT	CGTGCTGAATAATACCACTCACA
**VEGFA**	AGGGCAGAATCATCACGAAGT	AGGGTCTCGATTGGATGGCA
**GAPDH**	ACCACAGTCCATGCCATCAC	CACCACCCTGTTGCTGTAGCC
**SOST**	ACACAGCCTTCCGTGTAGTG	GGTTCATGGTCTTGTTGTTCTCC
**STIM-1**	AGTCACAGTGAGAAGGCGAC	CAATTCGGCAAAACTCTGCTG
**Fibronectin**	CAGTGGGAGACCTCGAGAAG	GTCCCTCGGAACATCAGAAA
**VEGFA**	AGGGCAGAATCATCACGAAGT	AGGGTCTCGATTGGATGGCA
**GRP78**	CATCACGCCGTCCTATGTCG	CGTCAAAGACCGTGTTCTCG

Supplementary Table S4. Primers used for Real-Time PCR
